# Distribution of *Plasmodium* species and assessment of performance of diagnostic tools used during a malaria survey in Southern and Western Provinces of Zambia

**DOI:** 10.1186/s12936-019-2766-2

**Published:** 2019-04-11

**Authors:** Lungowe Sitali, John M. Miller, Mulenga C. Mwenda, Daniel J. Bridges, Moonga B. Hawela, Busiku Hamainza, Elizabeth Chizema-Kawesha, Thomas P. Eisele, James Chipeta, Bernt Lindtjørn

**Affiliations:** 1 Centre for International Health, Faculty of Medicine, University of Bergen, Bergen, Norway; 2 Department of Biomedical Science, School of Health Sciences, University of Zambia, Lusaka, Zambia; 3 School of Medicine and University Teaching Hospital Malaria Research Unit (SMUTH-MRU), Lusaka, Zambia; 4 PATH Malaria Control and Elimination Partnership in Africa (MACEPA), National Malaria Elimination Centre, Ministry of Health, Chainama Grounds, Lusaka, Zambia; 5 National Malaria Elimination Centre, Ministry of Health, Chainama Hospital and College Grounds, Lusaka, Zambia; 6 Department of Tropical Medicine, Tulane University School of Public Health and Tropical Medicine, New Orleans, LA USA; 7 Center for Applied Malaria Research and Evaluation, Tulane University School of Public Health and Tropical Medicine, New Orleans, LA USA; 8 Department of Paediatrics and Child Health, University of Zambia School of Medicine, Lusaka, Zambia

**Keywords:** Malaria, Mixed infections, Non-falciparum infection, Rapid diagnostic tests, Zambia

## Abstract

**Background:**

Zambia continues to make strides in reducing malaria burden through the use of proven malaria interventions and has recently pledged to eliminate malaria by 2021. Case management services have been scaled up at community level with rapid diagnostic tests (RDTs) providing antigen-based detection of falciparum malaria only. Key to national malaria elimination goals is the ability to identify, treat and eliminate all *Plasmodium* species. This study sought to determine the distribution of non-falciparum malaria and assess the performance of diagnostic tests for *Plasmodium falciparum* in Western and Southern Provinces of Zambia, two provinces planned for early malaria elimination.

**Methods:**

A sub-set of individuals’ data and samples from a cross-sectional household survey, conducted during peak malaria transmission season in April and May 2017, was used. The survey collected socio-demographic information on household members and coverage of malaria interventions. Malaria testing was done on respondents of all ages using blood smears and RDTs while dried blood spots were collected on filter papers for analysis using photo-induced electron transfer polymerase chain reaction (PET-PCR). Slides were stained using Giemsa stain and examined by microscopy for malaria parasites.

**Results:**

From the 1567 individuals included, the overall prevalence of malaria was 19.4% (CI 17.5–21.4) by PCR, 19.3% (CI 17.4–21.4) by RDT and 12.9% (CI 11.3–14.7) by microscopy. Using PET-PCR as the gold standard, RDTs showed a sensitivity of 75.7% (CI 70.4–80.4) and specificity of 94.2% (CI 92.8–95.4). The positive predictive value (PPV) was 75.9% (CI 70.7–80.6) and negative predictive value (NPV) was 94.1% (CI 92.1–95.4). In contrast, microscopy for sensitivity, specificity, PPV, and NPV values were 56.9% (CI 51.1–62.5), 97.7% (CI 96.7–98.5), 85.6% (CI 80.0–90.2), 90.4% (CI 88.7–91.9), respectively. Non-falciparum infections were found only in Western Province, where 11.6% of *P. falciparum* infections were co-infections with *Plasmodium ovale* or *Plasmodium malariae*.

**Conclusion:**

From the sub-set of survey data analysed, non-falciparum species are present and occurred as mixed infections. As expected, PET-PCR was slightly more sensitive than both malaria RDTs and microscopy to detecting malaria infections.

## Background

*Plasmodium falciparum* is the major cause of malaria in Africa, while *P. vivax* is the most widely distributed species outside Africa [1], and in a few African countries, such as Ethiopia [2] and Uganda [3]. Compared with these two dominant species, *Plasmodium malariae* and *Plasmodium ovale* are significantly rarer and to a large extent are under-studied. *Plasmodium ovale* has been reported to be primarily distributed throughout sub-Saharan Africa [4]. *Plasmodium malariae* is found in tropical Africa where co-infections are sometimes encountered with *P. falciparum* [5].

Malaria remains a major public health problem in 91 countries worldwide, despite being preventable and treatable. It was linked to 216 million cases and 445,000 deaths in 2016, of which 90% were in sub-Saharan Africa [6]. Zambia has recorded a drop in malaria incidence from 407 cases per 1000 population in 2014 to 335 cases per 1000 population in 2015 [7] and it continues to make strides in reducing malaria cases through the use of proven and effective malaria interventions. It recently pledged to eliminate malaria altogether, through sustained universal coverage of vector control interventions, which include indoor residual spraying (IRS), distribution of long-lasting insecticide-treated nets (LLINs) and larval source management (LSM). Other important strategies include case management, health promotion, surveillance, and research [8]. Case management services are increasingly occurring at community level with standard rapid diagnostic tests (RDTs) providing antigen-based detection of falciparum malaria only and treatment with artemisinin-based combination therapy (ACT) for uncomplicated malaria coupled with injectable artesunate for severe cases. In addition, mass drug administration (MDA) has been included as an potential accelerator of the malaria elimination process [8].

Prompt and accurate case management of malaria infections is dependent on the performance of diagnostic tools. Readily available diagnostic tools include light microscopy of blood smears, RDTs, and molecular approaches such as polymerase chain reaction (PCR) and loop-mediated isothermal amplification (LAMP) [9, 10], although PCR is mostly used for research and not routine clinical diagnosis. Expert malaria microscopy remains an ideal diagnostic for malaria but due to a number of factors it cannot be used in all health facilities, hence the use of RDTs. RDTs are immunochromatographic tests that detect one or more of a range of antigens, namely histidine-rich protein 2 (HRP2), *Plasmodium* lactate dehydrogenase (pLDH) and aldolase [11]. Aldolase and pLDH are enzymes in the glycolytic pathway, while HRP2 is a water-soluble protein that is produced by asexual trophozoites and young gametocytes [12]. As HRP2 is produced exclusively during the asexual stages of the life cycle of *P. falciparum*, RDTs based on HRP2 detection are specific for *P. falciparum* [13], and are the only RDTs used in government health facilities in Zambia. RDTs are easy to use, do not require specialized training, can be performed in a clinic, health centre and hospital in the absence of electricity, and give results rapidly [14]. For these reasons, they have been widely adopted even at the lowest level in service delivery, the community level [15].

As with any diagnostic, there are limits to their utility and in some settings have shown poor sensitivity and specificity. For example, in a holo-endemic area of northern Tanzania, the sensitivity of the ParaHIT test was found to be 10.7% [16], while the sensitivity of the SD-Bioline test assessed in South Kivu Province of the Democratic Republic of the Congo was found to be 82.1% with a specificity of 92.0%, using microscopy as the standard [17]. The World Health Organization (WHO) recommended a threshold for sensitivity of > 95% and a threshold for specificity of > 90 [18].

As a country moves toward malaria elimination, it is crucial to ensure that all malaria cases irrespective of the infecting species are diagnosed and treated promptly. In Zambia, the distribution of *Plasmodium* species is not well defined. Despite this, HRP2-based RDTs are used for diagnosis in all facilities where microscopy is not available. This decision was based on information suggesting that 98% of malaria in Zambia was *P. falciparum,* 2% were *P. malariae,* while *P. vivax* is a rare infection [19], which may not have changed over the years. For example, a cross-sectional study conducted in high transmission areas in Eastern and Luapula Provinces revealed approximately 10.6% of all *P. falciparum* infections were co-infections with one or more other *Plasmodium* species. It is possible that in low transmission settings, the species distribution is different again, as species characterised by chronic infections (*P. malariae*) or dormant lifecycle stages (*P. vivax* and *P. ovale*) may constitute an increasing proportion of infections because of the chronic nature of *P. malariae* and the presence of the hypnozoite stages *P. ovale* and *P. vivax* [20]. These non-falciparum species potentially require additional interventions such as an anti-hypnozoite drug, e.g., primaquine.

## Methods

### Study design

A group of individuals were enrolled in across-sectional household survey conducted during peak malaria transmission season in April and May 2017, as part of ongoing efforts by the Zambia Ministry of Health, the PATH Malaria Control and Elimination Partnership in Africa (MACEPA) and other partners to evaluate malaria elimination efforts across Southern and Western Provinces.

The survey collected socio-demographic information on household members, coverage of malaria interventions, and additional social and behavioural information related to use of malaria interventions. As well as testing for malaria in the field with an RDT (SD Bioline malaria Ag pf, Standard Diagnostics Inc., Republic of Korea), a thick blood smear and a dried blood spot (DBS) was collected for analysis at the National Malaria Elimination Centre (NMEC) laboratory.

The sampling methods for each province were different due to historical studies and enumeration in the two provinces. In Southern Province, there was a pre-existing sampling frame used during a previously implemented MDA trial. In the trial sampling from the 10 districts along Lake Kariba, 52 households were randomly selected from each of the 60 health facility catchment areas. In Western Province, where there was no pre-existing household sampling frame, a two-stage cluster sampling with clusters selected using probability proportional to size (a standardized method from the country’s Malaria Indicator Survey) was used to select 25 households from 24 census-derived standard enumeration areas [21, 22]. All consenting or assenting individuals above 1 month of age (n = 6977) were enrolled in the two surveys. Those that were severely sick, were taken to clinic by survey staff, but information about them would be collected from the household respondent and no finger stick data would be collected.

With the help of OpenEpi software (Emory University, Rollins School of Public Health, USA) [23], ~ 300 samples were calculated using the prevalence of different species at 10.6% [34] rounding up to 11% for high transmission areas, and estimating 2% in low transmission areas, accuracy could be determined with 80% power and 95% confidence.

After excluding 41 clusters that were outliers by RDT prevalence, a total of 13 clusters were then randomly selected: 6 from Western (high RDT prevalence) and 7 from Southern (low RDT prevalence). All individuals from these clusters with complete survey data, including RDT and microscopy results, together with an identifiable DBS were selected for PCR speciation analysis (n = 1567), while those with insufficient blood and missing data were excluded. Due to time and cost of doing PCRs, all 6977 samples could not be analysed.

### Study area

**Fig. 1:**
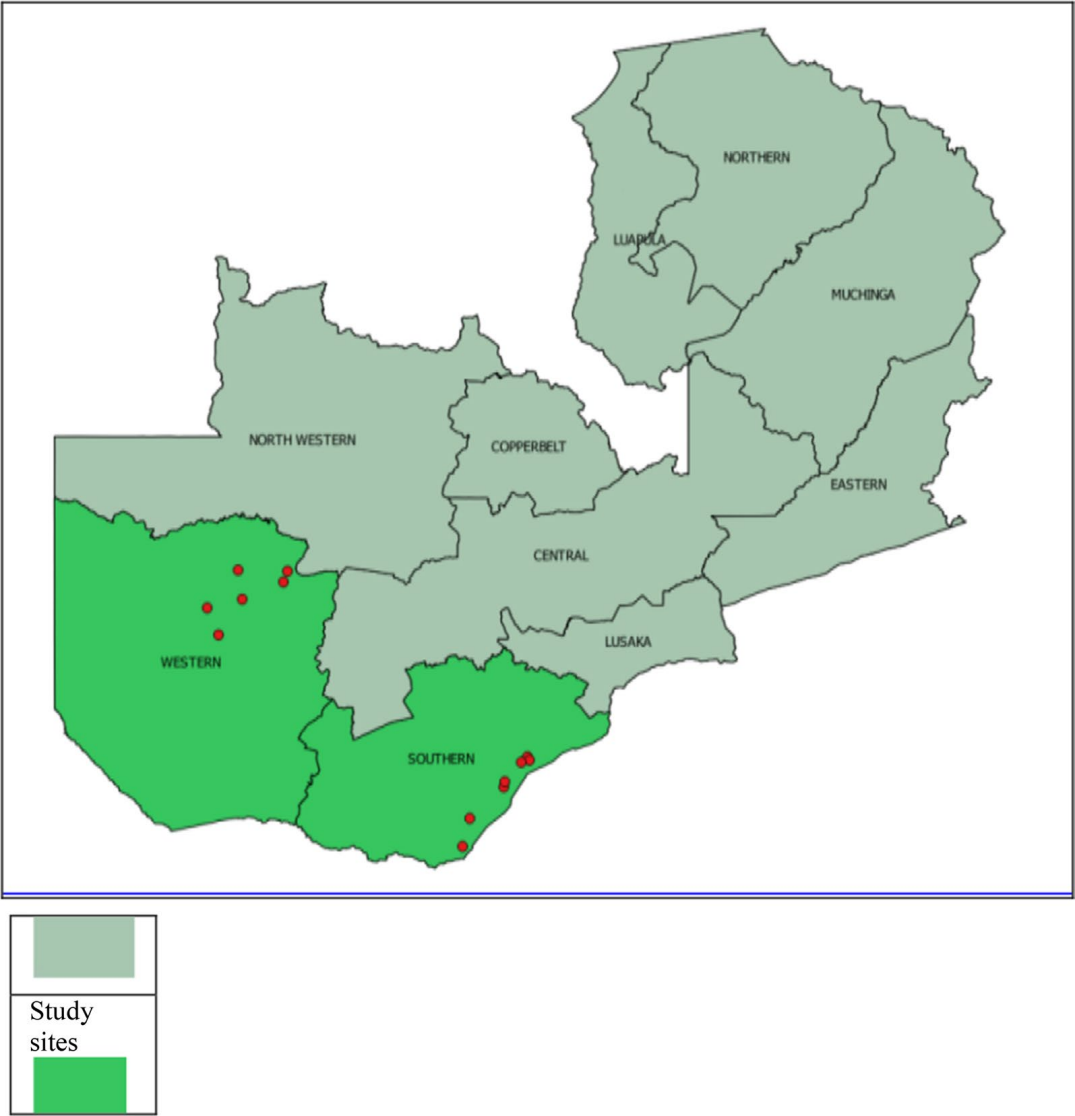
Map of Zambia showing location of Southern and Western Provinces. The study area is highlighted showing the red circles indicate the exact locations where samples were collected in the two provinces

The two provinces, Western and Southern, cover approximately 126,386 km^2^ (17% Western) and 85,823 km^2^ (11% Southern) of the total Zambia landmass, and are home to 902,974 (Western) and 1,589,926 (Southern) people, according to the 2010 population census. The Zambezi River flows through both provinces and the plains cover about 10% of the total area of Western Province. Tonga-speaking people in Southern and Lozi-speaking people in Western are the predominant ethnic groups [24, 25]. A map of Zambia in Fig. 1 shows the location of these two provinces. Malaria transmission varies greatly across these two provinces, with traditionally higher transmission intensity in Southern Province along Lake Kariba and in areas of Western Province around the swamps and wetlands in Luampa, Kaoma and Nkeyema districts and along the Zambezi River basin [21, 22, 26]. The rest of the areas away from water bodies have low transmission.

### Laboratory methods

#### Microscopy

Blood smears for microscopy were prepared in the field by trained biomedical scientists, air dried in a dust-free environment and stored in slide boxes. They were then transported to the NMEC where they were stained using 3% Giemsa for 45 min. The slides were examined independently by two experienced biomedical scientists.

#### DNA extraction

DNA was extracted from 6 mm (~ 13.8 µl whole blood) DBS punch(es)using a Qiagen DNA mini kit (Qiagen, Germany) and eluted in 100 µl. All RDT-positive samples were extracted alone, while RDT-negatives were extracted in pools of 10, and deconvoluted if positive.

#### PCR analysis

PET-PCR (real-time PCR technique), as previously described in 2013 by Lucchi et al. [27] was used to amplify *Plasmodium* 18S ribosomal RNA (see Table 1 materials for sequences). Briefly, 5 µl of DNA template (~ 0.7 µl whole blood) was amplified in a 20-µl reaction as follows: 95 °C for 15 min, followed by 45 cycles of 95 °C for 20 s and 60 °C for 40 s. Samples were analysed in duplicate and scored positive if both duplicates had a CT value < 40. The amplicon sizes were 109 bp *P. falciparum*, 137 bp *P. malariae,* 74 bp *P. ovale*, and 82 bp *P. vivax* [28, 29].

**Table 1:** Primers for species identification used for PET-PCR

Primer name	Sequence (5′–3′)
Original genus 18sFor	GGC CTA ACA TGG CTA TGA CG
Original genus FAM 18sRev	FAM-aggcgcatagcgcctggCTGCCTTCCT TAG ATGTGG TAG CT
Falciparum For	ACC CCTCGCCTG GTG TTT TT
Falciparum Rev	HEX-aggcggataccgcctggTCGG GCC CCA AAA ATA GGA A
*P. vivax* For	GTA GCC TAAGAAGGC CGT GT
*P. vivax* Rev	HEX-aggcgcatagcgcctggCCTGGGG GAT GAA TAT CTC TAC AGC ACT GT
*P. malariae* For	AAGGCAGTAACACCAGCAGTA
*P. malariae* Rev (based on dihydrofolate reductase-thymidylate synthase (DHFR-TS) gene)	FAM-aggcgcatagcgcctggTCCCATGAAGTTATATTCCCGCTC
*P. ovale* For	FAM-aggcgcatagcgcctggCCACAGATAAGAAGTCTCAAGTACGATATT
*P. ovale* Rev	TTGGAGCACTTTTGTTTGCAA

Table showing forward and reverse primers for species identification used in PET-PCT assay

### Statistical analysis

Demographic and laboratory data of participants’ records were analysed using Stata version 13 (College Station, Texas, USA). Fisher’s exact test for proportions was used to assess the association between variables and mixed and non-falciparum infection.

Diagnostic method performance was assessed against PCR as the gold standard as sensitivity [true positive (TP)/(TP + false negative (FN))], specificity [(TN)/(TN + false positive (FP))], positive predictive value (PPV) [TP/(TP + FP)]and negative predictive value (NPV) [TN/(TN + FN)]. The results were interpreted with 95% confidence intervals (CIs) GraphPad prism (GraphPad software Inc, San Diego, USA) was used to calculate Cohen’s Kappa agreement coefficient.

## Results

Table 2 shows the general and socio-demographic characteristics of the individuals included in this study. Age, gender, reported travel history, LLIN ownership were similar across both provinces, while IRS coverage was markedly higher in Southern (67.2%) compared to Western Province (31.6%).

**Table 2:** General and socio-demographic characteristics of participants

Characteristics	Southern; n = 1096		Western; n = 471	
	n (%)	CI	n (%)	CI
Gender				
Male	505 (46.1)	43.6–48.5	210 (44.6)	39.5–49.8
Female	591 (53.9)	51.5–56.4	261 (55.4)	50.2–60.5
Age of children (years)				
< 5	197 (18.0)	14.2–22.5	86 (18.3)	15.6–21.2
5–10	200 (18.3)	16.0–20.7	87 (18.5)	14.9–22.6
11–15	178 (16.2)	13.5–19.4	69 (14.7)	10.6–20.0
16–25	140 (12.8)	10.5–15.5	72 (15.3)	11.8–19.6
26–40	191 (17.4)	15.1–20.0	59 (12.5)	7.4–20.3
40–94	190 (17.7)	14.3–20.8	98 (20.8)	17.6–24.4
Travel history				
Yes	6 (0.5)	0.2–1.4	23 (4.9)	1.7–13.2
No	1089 (99.5)	98.6–99.8	445 (95.1)	86.8–98.3
Household sprayed				
Yes	730 (67.2)	52.4–79.1	149 (31.6)	8.7–69.1
No	357 (32.8)	20.8–47.6	322 (68.4)	30.9–91.2
ITN ownership				
Yes	673 (61.4)	38.8–80.0	269 (57.1)	29.2–81.1
No	13 (1.2)	0.1–9.7	8 (1.7)	0.3–8.6
Missing information	410 (37.4)	18.7–60.8	194 (41.2)	17.1–70.4
Cluster				
Average no of people/cluster	156.5		78.5	
Number of clusters	7		6	

The tables shows the general and socio-demographic characteristics of the participant in the two study areas

**Fig. 2:**
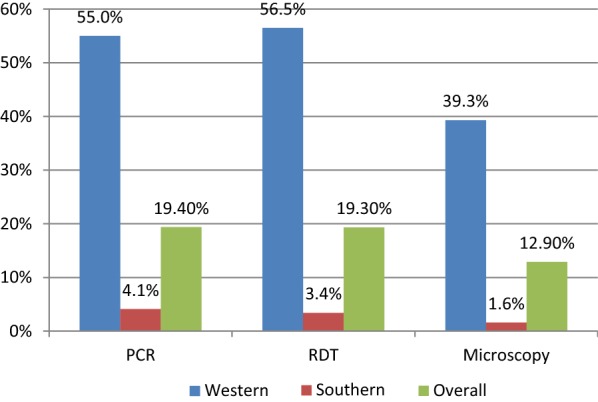
Prevalence of malaria among the participants by province

**Fig. 3:**
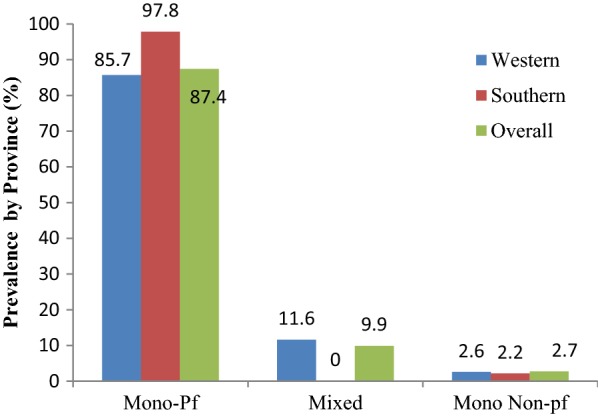
Prevalence of *Plasmodium falciparum*, mixed infection and mono non-falciparum infection by PET-PCR

Malaria prevalence determined by RDT or PCR was broadly similar across both provinces, while microscopy was significantly lower. In contrast, Western Province had a significantly higher prevalence of malaria as compared to Southern, 55 versus 4% by PCR (Fig. 2). The majority of all PCR-positive infections were *P. falciparum* mono-infections with 85.7 and 97.8% in Western and Southern, respectively. No mixed/co-infections were found in Southern Province, but 11.6% of all positives in Western had more than one species present (Fig. 3). A total of 8 non-falciparum mono-infections were also identified. Of these, 6 were *P. ovale* infections (exclusively from Western Province) and 2 *P. malariae* infections, one in each Province (Table 3).

**Table 3:** Differential species distribution by province

Province	Western		Southern		Overall	
	n	%	n	%	n	%
Pf only	222	85.7	44	97.8	266	87.5
Po only	6	2.3			6	2.0
Pm only	1	0.4	1	2.2	2	0.7
Pf and Pv	1	0.4			1	0.3
Pf and Po	26	10.0			26	8.6
Pf and Pm	3	1.2			3	1.0
Total	259		45		304	

The majority of the infections were *P. falciparum*, with (222/259) 85.7% and (44/45) 97.8% in Western and Southern, respectively. There were (30/259) 11.6% (95% CI 8.4–16.0%) mixed infections in Western, while none were observed in Southern Provinces. The combination observed in Western Province were Pf/Pv 1/259 (0.4%), Pf/Po 26/259 (10.0%). Pf/Pm 3/259 (1.2%)

Pf: *Plasmodium falciparum*; Pm: *P. malariae*; Po: *P. ovale*; Pv: *P. vivax*

### Sensitivity, specificity, positive, and negative predictive values of diagnostic tools

RDTs had sensitivity of 75.5% [230/304 (95% CI 70.4–80.4%)] and a specificity of 94.2% [1190/1263 (95% CI 92.8–95.4%)], with a PPV of 75.9% [230/303 (95% CI 70.7–80.6%)] and NPV of 1190/1264 [94.1% (95% CI 92.1–95.4%)]. The observed agreement percentage was 90.97%, and Cohen’s Kappa was 0.71 (CI 0.66–0.75). Microscopy was observed to have a sensitivity of 56.9% [173/304 (95% CI 51.1–62.5)], specificity of 97.7% [1234/1264 (95% CI 96.7–98.5)], PPV of 85.6% [173/202 (95% CI 80.0–90.2)] and an NPV of 90.4% [1234/1365 (95% CI 88.7–91.9)]. The observed agreement percentage was 89.79% and a Cohen’s Kappa of 0.63 (CI 0.57–0.68) (Table 4). Results from RDTs and microscopy against PCR showed a substantial measure of agreement.

**Table 4:** Performance of RDTs and microscopy compared to PCR results

	PCR			Sensitivity (95% CI)	Specificity (95% CI)	PPV (95% CI)	NPV (95% CI)
	Positive	Negative					
RDTs							
Positive	230	73	303	75.5% (70.4, 80.4)	94.2% (92.8, 95.4)	75.9% (70.7, 80.6)	94.1% (92.1,95.4)
Negative	74	1190	1264				
Total	304	1263	1567				

	PCR			Sensitivity (95% CI)	Specificity (95% CI)	PPV (95% CI)	NPV (95% CI)
	Positive	Negative					
Microscopy							
Positive	173	29	202	56.9% (51.1, 62.5)	97.7% (96.7, 98.5)	85.6% (80.0, 90.2)	90.4% (88.7, 91.9)
Negative	131	1234	1365				
Total	304	1264	1567				

PPN: positive predictive value; NPV: negative predictive value

Table 5 shows the sensitivity and specificity for RDTs when the standard is microscopy. The sensitivity was 81.2% [164/202 (95% CI 75.1–86.3%)] and the specificity was 89.8% [1226/1365 (95% CI 88.1–91.4)], with a PPV of 54.1% [164/303 (95% CI 48.3–59.8)] and an NPV of 97.0% [1226/1264 (95% CI 95.9–97.8)]. The observed agreement percentage was 88.70% and a Cohen’s Kappa of 0.0.59 (CI 0.53–0.64). A moderate measure of agreement was observed. Cohen’s Kappa was calculated using graph pad online calculate [30].

**Table 5:** Performance of RDTs compared with microscopy results

RDT	Microscopy			
	Positive	Negative	Total	
Positive	164	139	303	Se = 81.2% (75.1, 86.3), Sp = 89.8% (88.1, 91.4), Ppv = 54.1% (48.3, 59.8), Npv = 97.0% (95.9, 97.8)
Negative	38	1226	1264	
Total	202	1365	1567	

## Discussion

This study identified the presence of four *Plasmodium* species (*P. falciparum, P. ovale, P. malariae, P. vivax*) in Western Province and two (*P. falciparum, P. malariae*) in Southern Province. Interestingly, the diversity of parasite species found in a province broadly correlated to the malaria prevalence, i.e., the higher the prevalence the greater the number of species found. From these data alone, it seems clear that non-falciparum infections are under-reported due to the use of *P. falciparum*-specific RDTs and challenges in achieving high quality microscopy [31, 32].

Overall, 97% of all malaria infections contained *P. falciparum*, of which 89% were mono-infections and 11.6% co-infections and very few mono non-falciparum infections were identified (< 3% of all infections). These findings are in agreement with previous studies which reported 10.6% mixed infections and 88% *P. falciparum* [33], and are also close to 98% *P. falciparum* reported by Wolfe [34]. The findings suggest that transmission is occurring through a common vector population or that transmission of each species is occurring in the same geography/human population. It is well known that some mosquito species are capable of transmitting multiple parasite species, e.g. *Anopheles gambiae* is able to transmit all *Plasmodium* species [35] and is found throughout Southern and Western Provinces. While the vectorial capacity for transmission is present in both provinces, it is unclear how much non-*P. falciparum* transmission is local and how much is imported through travel [35]. Considering the numbers, it is entirely plausible that *P. malariae* in Southern Province is maintained through importation, while there is more robust local non-*P. falciparum* transmission in Western Province. No association was found between travel history and presence of non-falciparum infections however, only travel in the last month was recorded. Considering non-falciparum malaria infections may be chronic or dormant for long periods of time, it is not possible to determine the source of infections in this study. Genotyping of the parasite population may help dissect this relationship by defining the parasite population diversity and relatedness of different infections.

Often the non-falciparum species are under-reported or not identified due to a number of factors: the use of *P. falciparum*-specific RDTs as observed in approximately 63% of health facilities in Zambia [36] and microscopy-related challenges, such as inadequate experience and training of microscopists to identify parasites other than *P. falciparum* [31, 32]. Other factors include morphological changes induced by haemolysis hampering the identification of the species [31].

While evidence for *P. vivax* transmission exists [37], the majority of the Zambian population are expected to be resistant to *P. vivax* infections due to carrying the Duffy FyFy genotype [38]. It was, therefore, surprising to find *P. vivax*, albeit in only one person. There is evidence of low-level *P. vivax* endemicity in sub-Saharan Africa [39, 40]. Evidence from the Malaria Atlas Project (MAP) shows that Zambia’s neighbours, Democratic Republic of Congo, Namibia, Botswana and Angola, have cases of *P. vivax* [39].

The sensitivity value was below the 95% threshold for both RDTs and microscopy when compared to PCR, while specificity was above the recommended 90%. The findings of RDT sensitivity of 75.5% using PCR as gold standard is consistent with other publications on RDT sensitivity e.g., 88.6% in mainland Tanzania [41], 76.5% in Zanzibar [42] and 75.4% in Kisumu, Kenya [43].

In this study RDTs and microscopy had a relatively low PPV (< 90%), meaning that many positives were not infected, which in turn affects malaria morbidity, prevalence and incidence estimates [43]. Furthermore, treatment given to false positives is potentially costly and may lead to inaccurate perceptions of therapeutic failures [43]. A proportion of false positive individuals may reflect a recent resolved past infection due to HRP2 persistence. Conversely, a proportion of false negatives could be due to the functional loss of HRP2 expression, e.g., through a gene deletion as has been reported in neighbouring DRC [44]. Finally, the prozone effect in hyper-parasitaemic infections could also account for some of the false negatives [45, 46], although in this study the correlation between parasite density and false negatives was not assessed.

### Study limitations and strengths

Results of this study should be interpreted keeping some limitations in mind. Firstly, there was no similar pre-2017 analysis of species in Southern Province to indicate whether there was previously a higher level of mixed and non-falciparum species present. This makes it difficult to be certain if the elimination activities in the province are responsible for the low positivity rate. Secondly, these data cannot be generalized to the whole country, as this analysis is for two provinces, despite representing lower and higher transmission zones (low meaning an area with parasitaemia less than 5% and high meaning an area with parasitaemia above 15%), and the moderate transmission zone is not represented. Thirdly, an element of selection bias cannot be excluded as clusters with zero RDT prevalence were excluded. It is possible that the clusters may have had non-falciparum species leading to underestimation of the species. Finally, the study used secondary data and the authors have little or no control of RDT results, which may have affected the sensitivity and specificity results. Four samples from participants that were RDT-positive and PCR-negative, who were given treatment within 14 days, were included in the analysis. It is possible that the positive RDTs could have been due to lagging antigenaemia. Although on recalculation of sensitivity and specificity, new figures were within the same range as those previously calculated.

## Conclusion

There is a concern that other species could continue to drive transmission but remain undetected, as the RDTs currently used in Zambia detect only *P. falciparum*. While this study confirms that *P. falciparum* dominates, a not-insignificant 9.9% of these infections were mixed with another species. Encouragingly, where malaria is closest to elimination (i.e., in Southern Province), non-falciparum infections identified in this study were minimal (1 case). This may suggest that interventions that reduce *P. falciparum* transmission also impact non-falciparum species. It is important to expand surveillance activities to monitor non-falciparum infections and ensure this remains the case.

From the finding, recommendation made include: the national malaria programme consider increasing capacity to diagnose and detect non-falciparum species. This can be done through strengthening diagnosis quality assurance, increase access to functional microscopy, and provide refresher training in malaria microscopy for all laboratory staff, and in addition, introduce Pan RDTs (RDTs able to diagnose *P. falciparum* and non-falciparum species).

## Abbreviations

AL: artemether–lumefantrine
HRP2: histidine-rich protein 2
DBS: dried blood spot
DNA: deoxyribonucleic acid
ITN: insecticide-treated net
IRS: indoor residual spraying
LLINs: long-lasting insecticide-treated nets
LSM: larval source management
NPV: negative predictive value
NMEC: National Malaria Elimination Centre
NMCP: National Malaria Control Programme
MACEPA: Malaria Control and Elimination Partnership in Africa
MDA: mass drug administration
MIS: Malaria Indicator Survey
PCR: polymerase chain reaction
PET-PCR: photo-induced electronic transfer-polymerase chain reaction
pLDH: parasite lactate dehydrogenase
PPV: positive predictive value
RDTs: rapid diagnostic tests
